# Community treatment orders in England: review of usage from national data

**DOI:** 10.1192/bjb.2017.33

**Published:** 2018-06

**Authors:** Susham Gupta, Elvan U. Akyuz, Toby Baldwin, David Curtis

**Affiliations:** 1 East London NHS Foundation Trust, UK; 2 North and East London NHS Foundation Trust, UK; 3 University College London Genetics Institute, UK

## Abstract

**Aims and method:**

Community treatment orders (CTOs) have been in used in England and Wales since November 2008; however, their effectiveness has been debated widely, as has the question of which methodology is appropriate to investigate them. This paper uses national data to explore the use of CTOs in England.

**Results:**

About 5500 patients are subject to CTOs at any one time. Each year, ~4500 patients are made subject to a CTO each year and ~2500 are fully discharged, usually by the responsible clinician; fewer than half of CTO patients are recalled, and two-thirds of recalls end in revocation. The low rate of CTO discharges by mental health tribunals (below 5%) suggests that they are not used inappropriately.

**Clinical implications:**

The introduction of CTOs in England has coincided with a reduction in psychiatric service provision due to the economic downturn. Pressures on services might be even more severe if patients currently subject to CTOs instead needed to be detained as in-patients.

**Declaration of interest:**

None.

## Background

Community treatment orders (CTOs) were introduced into legislation for England and Wales by the Mental Health Act (MHA) 2007. They can be applied to a patient who is already subject to a section of the MHA which makes them liable to detention for treatment. The patient is discharged from the treatment section on to a CTO, which broadly means that they need to comply with a set of specified conditions that may include accepting prescribed treatment. The responsible clinician has the power to recall a patient to hospital. If the CTO is revoked, the treatment section is reinstated. CTOs are initially valid for 6 months but can be extended indefinitely. They are subject to scrutiny by independent first-tier mental tribunals (Mental Health), or MHTs, which can discharge the patient from the CTO if the grounds for its use, as laid out in the MHA, are deemed not to be met. Following the introduction of CTOs, uptake was initially higher than some had predicted, and over the past few years usage seems to be fairly stable.^1^^–^^3^ The attitude of many clinicians to CTOs has been favourable,^4^ but they have also been the subject of controversy.^4^^,^^5^ The power to coerce patients to accept treatment in the community clearly represents a conflict between the principles of respecting autonomy and of preventing harm to the patient and/or others, and thus between providing the ‘least restrictive’ treatment option balanced against ‘preventive’ principles.^6^ The difficulties in resolving this conflict satisfactorily have resulted in significant variations in the nature and implementation of these orders in different countries. Factors which could reduce the usage of CTOs may include a reluctance to use coercive measures, objections from patients' advocates, concerns about liability, operational aspects and the lack of a strong evidence base.^7^

CTOs are typically indicated for psychiatric patients, usually with a diagnosis of psychosis, presenting with a ‘revolving door’ pattern of admissions secondary to poor treatment adherence and poor engagement with psychiatric services.^8^ Although a main aim of CTOs is to reduce readmissions by preventing relapse, another motivation might be to attempt to improve quality of life for patients and their careers. The responsible clinician devises a set of conditions appropriate to the individual patient in the CTO. The responsible clinician has the discretion to recall the patient, initially for a 72-h period, for further treatment if deemed necessary. During this recall period, a clinical decision has to be made as to whether the CTO should be revoked, which leads to the patient returning to being subject to the original hospital treatment section from which they were discharged on to the CTO. Alternatively, they may receive some brief intervention and be allowed to return to the community, or they may be informally admitted while continuing to be subject to the CTO. The responsible clinician has considerable discretion as to when to exercise the power of recall. Some clinicians may aim to recall patients promptly, with the aim of quickly re-establishing treatment and preventing further deterioration, possibly without needing to revoke the CTO. Others may prefer to wait longer, in the hope that things will improve without having to subject the patient to a measure which may seem overly coercive or even punitive. A recent follow-up study found that fewer than half the patients subject to CTOs are recalled, with about a fifth being recalled multiple times.^9^ In that study, half of these recalls were due to deterioration in clinical condition and about 70% of recalls resulted in revocation.

## Effectiveness of CTOs

If CTOs were effective and were used effectively, they might lead to an overall reduction in requirement for in-patient treatment and a decline in the number of patients detained on treatment sections.^10^ Two older American randomised controlled studies (RCTs) of similar measures failed to find clinical benefits, but it has been argued that they had significant methodological drawbacks, for example, in that they excluded high-risk patients.^11^^,^^12^ Subsequent American studies have claimed to demonstrate benefits, especially when considered as part of a wider public health system involving the criminal justice system.^7^ Given the difference in psychiatric service provision, these studies may have limited applicability to the UK. The OCTET study, a RCT which was carried out soon after the introduction of CTOs, did not find clinical benefits.^13^ However, this study has been criticised as having significant methodological problems, such as again excluding high-risk patients and the fact that the CTOs were only used for a brief period of time.^14^^,^^15^ Small naturalistic UK clinical studies using before and after methodologies have reported positive outcomes.^16^^–^^18^ Swartz and Swanson (2015)^15^ suggested that RCTs may not be the best way to study these complex tools, and that larger, naturalistic studies may be more appropriate. A Care Quality Commission (CQC) 2009/10 report claimed that a third of CTO patients in England did not have a reported history of non-adherence or disengagement.^19^

Figures from the Mental Health Minimum Data Set show that the majority of people on CTOs are of working age, and more than twice as many are male than female; however, in the 65 and over age group, more women than men are on CTOs.^20^ The descriptive data indicate that patients are typically male and around 40 years of age, with a long history of schizophrenia-like or serious affective illness, previous admissions, poor medication adherence, aftercare needs, the potential for violence and displaying psychotic symptoms, especially delusions, at the time of the CTO.^6^ CTO usage is more prevalent in urban areas.^21^

## Method

We examined national data on aspects of CTO usage alongside that of other provisions of the MHA and information on psychiatric service provision. National data from the annual reports of Digital NHS (previously the Health and Social Care Information Centre) and Mental Health Reports of the CQC (the independent regulator of health and social care in England since 2009/10) were studied to look at the trends in implementation of CTOs. Thus, although CTOs are used in both England and Wales, the results we report are only for England.

## Results

All the figures quoted in the results below and accompanying tables were extracted from the NHS Digital report: *In-Patients Formally Detained in Hospitals Under the Mental Health Act 1983, and Patients Subject to Supervised Community Treatment: 2015/16, Annual Figures*.^21^

As shown in Table 1, the annual rate of new CTOs is fairly stable at around 4500, with the number of patients subject to CTOs at any one time being around 5400. The annual number of discharges from CTOs has steadily increased over 5 years from 1712 (2011/12) to 2575 (2015/16), and each year somewhat fewer than half of all patients subject to a CTO are discharged.

**Table 1:** Annual numbers of patients in England who are subject to CTOs at any one time, along with the number of new CTOs initiated and numbers and percentages of patients discharged from CTOs

Year	2011/12	2012/13	2013/14	2014/15	2015/16
Number of patients subject to CTOs	4764	5218	5365	5461	5426
Number of new CTOs	4220	4647	4434	4564	4361
Number of CTO discharges	1712	2162	2230	2491	2575
Number of discharges as percentage of number of patients subject to CTO, %	35.9	41.4	41.6	45.6	47.5

As shown in Table 2, there are about 45 recalls per year for every 100 patients subject to a CTO (HSCIC 2015/16). Over the past 5 years, the average rate of revocation following such recalls is 65%, albeit with a fair degree of variation between years, with absolute numbers fluctuating between 1000 and 1500. Table 3 shows that, including these revocations, around 9000 patients are detained under Section 3 each year. Given that about 4500 patients are discharged on a CTO annually, it seems that about half of patients detained under Section 3 will be discharged on a CTO.

**Table 2:** Annual number of recalls and numbers of recalls per 100 patients subject to a CTO

Year	2011/12	2012/13	2013/14	2014/15	2015/16
Number of recalls from CTO	2082	2272	2316	2369	2294
Recalls per 100 CTO patients	48	44	43	43	42
Number of revocations	1469	1509	1401	1427	1557
Percentage of recalls resulting in revocation, %	70.6	66.4	60.5	60.2	67.9

(Note that some patients may be recalled more than once.) Also shown are the number of revocations and the percentage of recalls that result in revocation.

**Table 3:** Annual number of patients newly detained under Section 3 (S3) of the MHA, number of CTO revocations and the sum of these two numbers

Year	2011/12	2012/13	2013/14	2014/15
Non-CTO Section 3	7701	7776	7481	7690
Revocations from CTO	1469	1509	1401	1427
Total	9170	9285	8882	9117

Table 4 shows that each year patients make 3000–4000 applications to MHTs to be discharged from CTOs. Most applications proceed to a hearing, and the proportion of MHT hearings resulting in discharge was running at 4–5%, falling to 3.3% in 2015/16 (CQC 2012/13, CQC 2013/14, CQC 2014/15, CQC 2015/16). These rates are not dissimilar to the rates for discharge from hospital treatment sections (Section 3 and Section 37) of 4.4% in 2013/14, and less than that for all sections, which was 8.9% (CQC 2013/14). The application may not proceed to a hearing if it is withdrawn by the patient or if the responsible clinician themselves discharges the CTO. Of all patients subject to a CTO in a given year, the percentage discharged by a MHT is around 2.5–3.5%. The low rates could partly be explained by the higher proportions of automatic referrals made to the tribunal by hospital managers, as opposed to applications made by patients.

**Table 4:** Annual numbers of applications to MHTs for discharge from CTO, numbers of hearings, and numbers and percentages of hearings resulting in discharge by the MHT

Year	2011/12	2012/13	2013/14	2014/15	2015/16
Patients subject to a CTO	4764	5218	5365	5461	5426
Applications to MHT	3901	4211	4431	4349	4317
MHT hearings	3272	3169	3550	3629	3942
MHT discharges	161	132	185	165	132
Percentage of hearings resulting in discharge, %	4.9	4.2	5.2	4.5	3.3
Percentage of all CTO patients discharged by MHT, %	3.4	2.5	3.4	3.0	2.4

Also shown is the percentage of discharges by MHT of all patients subject to a CTO.

The past few years have seen a 17% reduction in the number of in-patient beds for people needing care for mental health problems: from 26 448 in 2008/09 to 21 949 in 2012/13.^22^ HSCIC data (2015) show that annual patient contact numbers have fallen significantly, while patient numbers are increasing.^23^ The same report also shows that the greatest fall has been for assertive outreach services (more than 20%) and general psychiatric, substance misuse and forensic services (around 15% each). By contrast, contact with criminal justice liaison and diversion services saw the greatest increase in contacts (36.2%), while contact with psychiatric liaison increased by almost 28%.

## Discussion

The rate of CTO use is about 10 per 100 000 of the population, which puts it in the low to moderate range by international comparison with similar provisions for compulsory treatment in the community. Australia and New Zealand and some parts of the USA have much higher rates, whereas rates are much lower in Canada and New York.^24^

The pattern of usage of CTOs seems to be fairly stable in the context of ongoing reductions in psychiatric service provision. A CQC report claimed that the powers were being applied ‘preventatively beyond those for whom they were primarily designed’.^19^ However, we note that the rate of discharge by MHTs is low and possibly falling, indicating that these independent tribunals do not seem to regard CTOs as being used inappropriately.

The number of recalls is nearly half the total number of CTOs, and somewhat fewer than a third of CTOs are ended by revocation. Again, the fact that such a large proportion of patients subject to CTOs end up requiring readmission might be taken as an indicator that CTOs are largely being used appropriately, in an attempt to provide treatment in the community to patients who would otherwise remain in hospital. It is not possible to tell whether the number of revocations could be reduced if patients were recalled more promptly, at an earlier stage in their relapse, or whether the revocations represent a group of patients who are intrinsically difficult to maintain in the community. It would be helpful to investigate this aspect of clinical practice.

It is difficult to know the extent to which the use of CTOs has allowed psychiatric services to continue to function with reduced bed provision. Of course, many would argue that psychiatric services are in fact not functioning at an acceptable level, and the rise in contacts with the criminal justice system could be taken as evidence of this. The MHT will uphold a CTO only if it feels that the CTO is required for the patient to continue to accept treatment. Clinically, adherence to treatment is aimed at preventing relapse and hospital admission among ‘revolving door’ patients, and has wider significance for psychiatric in-patient service provision.

Overall, the success or failure of CTOs hinges on their appropriate application and implementation. There may well be large differences in practice between services and individuals; it would be helpful to explore these systematically and, if possible, relate them to outcome measures. Large numbers of patients are subjected to this provision, so it would seem sensible to take whatever steps possible to see that it is used effectively.

## About the authors

**Susham Gupta** is a Consultant Psychiatrist at East London NHS Foundation Trust, UK; **Elvan U. Akyuz** is a Consultant Psychiatrist at North and East London NHS Foundation Trust, UK; **Toby Baldwin** is a Specialty Doctor at East London NHS Foundation Trust, UK; and **David Curtis** is Honorary Professor of Psychiatry at University College London Genetics Institute and the Centre for Psychiatry, Barts and the London School of Medicine and Dentistry, London, UK.
